# Multiparametric Evaluation of Post-MI Small Animal Models Using Metabolic ([^18^F]FDG) and Perfusion-Based (SYN1) Heart Viability Tracers

**DOI:** 10.3390/ijms222212591

**Published:** 2021-11-22

**Authors:** Tomasz Jan Kolanowski, Weronika Wargocka-Matuszewska, Agnieszka Zimna, Lukasz Cheda, Joanna Zyprych-Walczak, Anna Rugowska, Monika Drabik, Michał Fiedorowicz, Seweryn Krajewski, Łukasz Steczek, Cezary Kozanecki, Zbigniew Rogulski, Natalia Rozwadowska, Maciej Kurpisz

**Affiliations:** 1 Institute of Human Genetics, Polish Academy of Sciences, 60-479 Poznan, Poland; tomasz.kolanowski@igcz.poznan.pl (T.J.K.); agnieszka.zimna@igcz.poznan.pl (A.Z.); natalia.rozwadowska@igcz.poznan.pl (N.R.); 2 Biological and Chemical Research Centre, Faculty of Chemistry, University of Warsaw, 02-089 Warsaw, Poland; wargocka.w@gmail.com (W.W.-M.); lcheda@chem.uw.edu.pl (L.C.); rogul@chem.uw.edu.pl (Z.R.); 3 Department of Mathematical and Statistical Methods, Poznan University of Life Sciences, 60-637 Poznan, Poland; joanna.zyprych@gmail.com; 4 Institute of Human Biology and Evolution, Faculty of Biology, Adam Mickiewicz University, 61-614 Poznan, Poland; anna.rugowska@amu.edu.pl; 5 Mossakowski Medical Research Institute, Polish Academy of Sciences, 02-106 Warsaw, Poland; monika.angelika.drabik@gmail.com (M.D.); mfiedorowicz@imdik.pan.pl (M.F.); 6 Synektik S.A., 00-728 Warsaw, Poland; skrajewski@synektik.com.pl (S.K.); lsteczek@synektik.com.pl (Ł.S.); ckozanecki@synektik.com.pl (C.K.)

**Keywords:** fluorine radiotracers, SYN1, [^18^F]-FDG, FDG, positron emission tomography–computed tomography (PET/CT), molecular profiling after myocardial infarction, RNAseq of the heart tissue

## Abstract

Cardiovascular diseases (CVD), with myocardial infarction (MI) being one of the crucial components, wreak havoc in developed countries. Advanced imaging technologies are required to obtain quick and widely available diagnostic data. This paper describes a multimodal approach to in vivo perfusion imaging using the novel SYN1 tracer based on the fluorine-18 isotope. The NOD-SCID mice were injected intravenously with SYN1 or [^18^F] fluorodeoxyglucose ([^18^F]-FDG) radiotracers after induction of the MI. In all studies, the positron emission tomography–computed tomography (PET/CT) technique was used. To obtain hemodynamic data, mice were subjected to magnetic resonance imaging (MRI). Finally, the biodistribution of the SYN1 compound was performed using Wistar rat model. SYN1 showed normal accumulation in mouse and rat hearts, and MI hearts correctly indicated impaired cardiac segments when compared to [^18^F]-FDG uptake. In vivo PET/CT and MRI studies showed statistical convergence in terms of the size of the necrotic zone and cardiac function. This was further supported with RNAseq molecular analyses to correlate the candidate function genes’ expression, with *Serpinb1c*, *Tnc* and *Nupr1*, with *Trem2* and *Aldolase B* functional correlations showing statistical significance in both SYN1 and [^18^F]-FDG. Our manuscript presents a new fluorine-18-based perfusion radiotracer for PET/CT imaging that may have importance in clinical applications. Future research should focus on confirmation of the data elucidated here to prepare SYN1 for first-in-human trials.

## 1. Introduction

Fast, reliable and accessible diagnostics of heart function impairment is one of the most crucial elements of the cardiovascular diseases (CVDs) treatment process. CVDs are responsible for 17.9 million deaths yearly, which has made them the leading cause of death worldwide for over two decades now [1]. Out of these, ischemic heart disease causes 8.9 million deaths annually (data from 2019), and the number increased by 2 million cases from the beginning of the century [2]. This means that despite a significant effort to treat and diagnose heart diseases, ischemic cardiomyopathy (ICM)—a condition of heart muscle malfunction caused by ischemic heart disease/myocardial infarction (IHM/MI)—is still a major issue worldwide.

To contain this problem, a holistic approach has been undertaken, including prevention and development of new therapeutic approaches, but even in developed countries, easy access to fast and reliable heart imaging diagnostic is still a challenge.

The most widespread, advanced imaging techniques include Cardiac Magnetic Resonance (CMR) and Positron Emission Tomography combined with Computer Tomography (PET/CT). Differences between these methods have been extensively discussed [3], however, PET/CT shows major advantages in terms of reliability (better scan resolution), robustness (scan time counted in seconds), accessibility (approximately 100x shorter scan time, therefore increased throughput) and thus is preferred by patients (single breath hold). To functionalize the diagnostic values of the PET/CT, a contrast agent is applied. There are several types of isotope-based PET/CT contrast agents currently used in the clinics. This includes isotopes of ^82^Rb, ^15^O, and ^13^N with half-life times of 75 s, 2 min, and 10 min, respectively [4].

Currently, fluorine-18 is gaining popularity as an alternative because of its half-life of 110 min and its well-known chemistry [5]. The development of radiotracers based on the fluorine-18 isotope is becoming a priority due to the advantages of fluorine chemistry, but also because of the many disadvantages that the perfusion radiotracers used to date have suffered from. The mentioned half-time and photon energy provide lab technicians with a detailed timing of experiments compared to the other used isotopes. In addition, the easy availability of fluorine-18 from cyclotrons reduces the need for advanced devices onsite, as a much longer half-life allows the distribution of the isotope or ready-made compounds to distances from the cyclotron research and medical units, while the aforementioned ^82^Rb requires the purchase of expensive ^82^Sr/^82^Rb generators, and with ^15^O and ^13^N availability of the cyclotron on site [6].

When imaging with radioactive isotopes, the metabolic or perfusion capacity of the heart might be assessed, depending on the marker type. An example of the widespread metabolic radiotracer is 2-[^18^F]fluoro-2-deoxyglucose ([^18^F]-FDG) [7]. A valuable supplement to metabolic imaging is perfusion tests, which most often use radiotracers based on the technetium isotope. The common operating principle of perfusion radiotracers is that they are intended for intravenous administration so that they reach and accumulate in the target tissues. The amount of accumulated compound should be directly proportional to the blood supply. Depending on the radiotracer used, imaging may take from several minutes to several hours. Adequate time, dose and energy are crucial to ensure proper biodistribution of the compound, removal from the bloodstream and for obtaining high-quality images. Currently, the scientific effort is focused on the synthesis of fluorinated radiotracers targeted to assess the heart perfusion state. Fluorine-based cardiac perfusion radiotracers enable PET imaging as an alternative to SPECT assessment, and do not require the purchase of expensive generators or onsite synthesis. The most thoroughly studied compound for medical use is Flurpiridaz. Flurpiridaz is the latest generation ^18^F-labelled PET radiotracer for cardiac perfusion imaging, and is structurally an analogue of the insecticide pyridaben, a known inhibitor of NADH [8]. Besides Flurpiridaz, there are several other compounds currently under active development for potential clinical use with a similar or very similar mechanism of action [9]. Nevertheless, they all share some disadvantages, including ambivalent clinical evidence of effectiveness resulting in nonacceptance by FDA/EMA. Therefore, the screening and searching for more clinically accurate compounds to assess heart function is still ongoing.

Another issue is the lack of reliability of the imaging techniques when considering molecular changes within the heart that are the hallmarks of the deep-tissue damage during ischemia. Evaluation of several protein levels is commonly used in the early phases of ischemic damage diagnostics, but they are not stable in the long term and are only indirect estimates of the heart muscle impairment (only cardiac troponins levels remain elevated up to 2–3 weeks after AMI). Less is known about the direct molecular changes in the myocardium when compared with advanced imaging. Differences between function in metabolically impaired myocardium and areas with deep disruption of the tissue structure are addressed by “late-enhancement” imaging, although this relies on loss of activity, and the correlation between signal intensity and direct molecular expression changes in the heart muscle is rarely monitored.

Here, we have been assessing the new type of PET/CT perfusion contrast agent—SYN1 radiotracer (formerly known as CAD-SK-FMO-011) in the perfusion imaging of normal and postinfarction heart in mice.

SYN1 is an innovative myocardial perfusion imaging (MPI) agent and the most common imaging tool for noninvasive ischaemia evaluation in patients with suspected coronary artery disease (CAD). The SYN1 tracer has several advantages when compared to other clinically used compounds, including the half-life of the radioisotope (^18^F) ensuring stable signal and safety to the patients when compared with other isotopes in use (incl. ^201^Tl, ^82^Rb, ^13^N) [8,9,10,11]. SYN1 combines a reasonable half-life of 110 min and the positrons of the lowest energy.

The SYN1 and [^18^F]-FDG PET–CT scans are complementary to each other, and they are used to measure different parameters of the diagnosed heart muscle. SYN1 is recognized as a perfusion tracer, which shows delivery of blood to a capillary bed in tissue, while [^18^F]-FDG is a viability tracer used to determined which regions of the myocardium are still alive and will benefit from revascularization. Radiopharmaceuticals employed in our study have been administered in submolecular amounts, thus their potential toxicity is mainly related to the interaction of ionizing radiation with the body. Considering the low activity of the administered preparations, no negative effects of radiation on the test organism should be expected. The amount of the chemical impurities is also on a low level—within the range of tens of µg/mL—which also should not produce a negative effect.

In our study, the mice myocardial infarction model (LAD ligation) was used to compare SYN1 to other two gold standard imaging systems—[^18^F]-FDG PET/CT and MRI (magnetic resonance imaging/CMR—cardiac magnetic resonance). We estimated the staining consistency of the metabolic radiotracer [^18^F]-FDG with the perfusion radiotracer (SYN1) with impaired cardiac function due to myocardial infarction when confronted with MRI heart performance indicators. Furthermore, in this study, we defined extensive molecular profiling of the tissue expression changes after MI and selected several marker genes, confirming the efficiency of their use in cardiac tissue ischemic injury. By combining molecular profiling with imaging technique data, we attempted to correlate imaging techniques’ reliability. Finally, we tested the biodistribution of SYN1, exploiting the possibility for its clinical use.

## 2. Results

### 2.1. SYN1 Distribution Correlates with the Size of the Infarction

**Figure 1:**
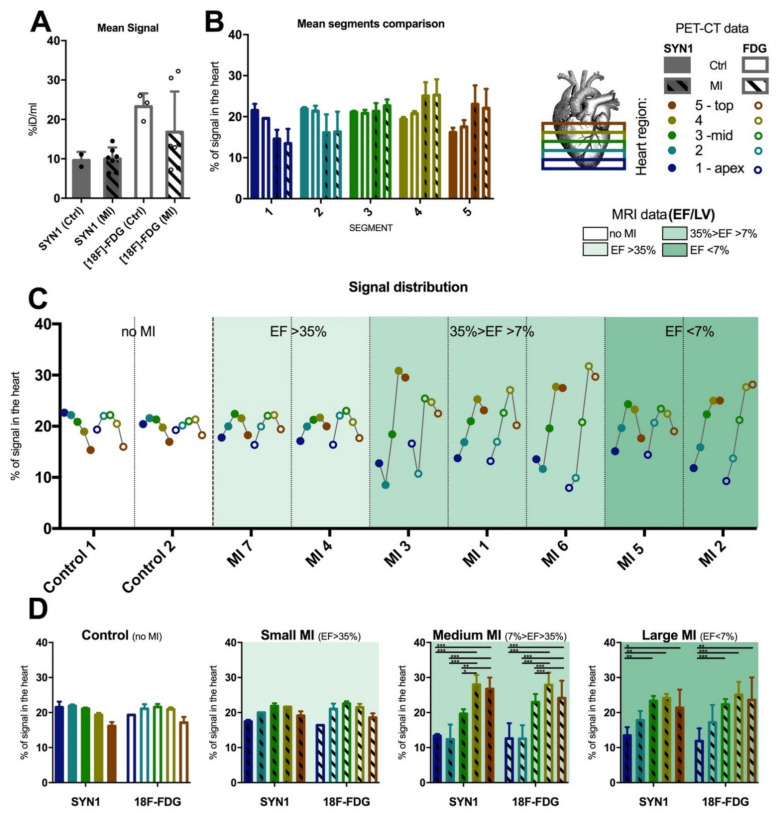
Comparison of the perfusion-based SYN1 vs. metabolic [^18^F]-FDG signalling using PET/CT with inclusion of MRI results in MI mice and the control group. A high signal-to-signal variation could be observed in the batch analysis of MI-affected groups with both radiotracers – individual and mean signal values has been presented (**A**). Hearts (and normalized signals) were divided into five isovolumetric segments with most differences observed in segments 4 and 5 when compared to the control (**B**). Representation of each segment of the individual heart showing heterogeneity of signals in MI affected hearts (patterned bars) compared to control (nonpatterned bars) (**C**). The highest heterogeneity was observed in the case of medium-sized MI (7% < LV EF < 35%). (**D**) Results have been grouped based on the MI size as evaluated by MRI. These were: small MI (LV EF > 35%), medium MI (7% > LV EF > 35%), large MI (LV EF < 7%) and nonaffected control; each bar represents different heart segments from 1 to 5 (from left to right). Statistically significant differences in the signal distribution in the heart have been observed in medium and large MI groups. In all cases, groups have been marked according to the legend with full bars marking SYN1-based measurements and empty-interior bars indicating [^18^F]-FDG measurements. In both cases, bars with the slash-like pattern are considered MI groups while nonpatterned bars are the control groups. *p*-value: * <0.033; ** <0.002; *** <0.001.

In the case of using SYN1 or [^18^F]-FDG in the study groups, the PET/CT static measurements were performed 30 min (SYN1) or 60 min ([^18^F]-FDG) after intravenous administration into the tail vein. Due to the large variability of the results in the myocardial infarction (MI) group, comparison of the SYN1 and [^18^F]-FDG uptakes (%ID/mL) in the heart of MI and control mice did not show significant differences (Figure 1A), Nevertheless, obtained images significantly indicated a loss of uptake of compounds in the periapical area (Figure S1). We then divided each analysed heart into five segments equilibrated in terms of the total number of slices; with segment 1 starting at the apex and segment 5 ending at the left ventricle top (see Figure 1B and the figure legend). Defined heart segments have shown a reverted trend with increased signal strength in the lower parts of the heart tissue (segments 1 and 2), while in MI groups the signal was increased in higher parts of the organ (segments 4 and 5) suggesting more metabolically active and perfused tissue present in these areas (Figure 1B), with the signal in the MI group remaining heterogenous. To define precise signal distribution, we decided to perform individual analysis of each heart (Figure 1C). Mice have been organised according to the percentage of ejection fraction of the left ventricle descending to show heterogeneity of the signals in both evaluated radiotracers (SYN1 and [^18^F]-FDG). Clearly, the MI of medium size showed the most significant differences in normalised signal distribution, with segments 1 and 2 most affected when compared with segments 3, 4, and 5 (Figure 1D). These results proved that the tested radiotracer SYN1 had similar efficiency and capability for discrimination between the healthy and diseased tissue compared to reference radiotracer—[^18^F]-FDG.

### 2.2. Evaluation of the Gene Expression Profile in the Heart after Myocardial Infarction

After functional evaluation of the radiotracers, expression analysis of the gene profile was performed in which the myocardial infarction group was compared with the healthy control. As we aimed to evaluate only confirmed, functional RNAs that might have a direct influence on clinical research, only RNAs with known or predicted functions that have been up- or down-regulated (*p* < 0.01; −2 > logFC > 2) and which represented at least 100 reads in the group of which expression was markedly different than the background, were taken into account. Such data filtering allowed us to avoid misleading results of high expression changes in doubtful RNA targets or in RNAs that might have been represented by less than several full-length transcripts in the sample. Among differentially expressed genes, 35% of them have been marked as extracellular and 23% as localized in the nucleus (Figure S2). In terms of upregulated pathways HIF-1, PI3K-Akt were overrepresented, with the Integrin signalling pathway presenting the most downregulated genes (Figure S2).

**Figure 2:**
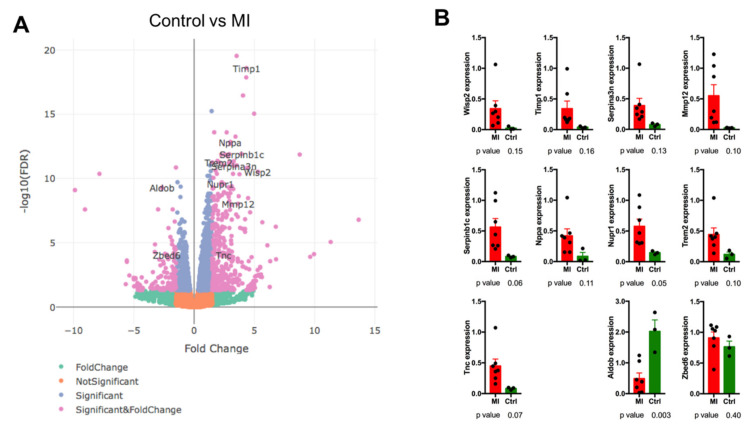
Analysis of expression profile in control vs. stable MI group. (**A**) A volcano plot of up- and down-regulated genes with genes of particular interest marked. RNAseq analysis was performed using following criteria: *p* < 0.01; −2 > logFC > 2; only RNA targets with known/predicted function; minimal prevalence for target gene in the sample with higher expression = 100 reads (of 75 bp each). (**B**) Confirmation of expression in PET/CT group of mice using RNAseq. All results have been normalized to GAPDH expression. Dots represent particular samples, while bars represent mean ± SD. *p*-values ≤ 0.1 were considered to be significant.

Among genes upregulated in the MI group we have found WNT1 Inducible Signaling Pathway Protein 2 (*Wisp2*), Tissue Inhibitor Of Metalloproteinases 1 (*Timp1*), Serpin Family B Member 1 (*Serpinb1c*), Matrix Metallopeptidase 12 *(Mmp12*), Serpin Family A Member 3 *(Serpina3n*), Natriuretic Peptide A (*Nppa*), Nuclear Protein 1, Transcriptional Regulator (*Nupr1*), Triggering Receptor Expressed On Myeloid Cells 2 (*Trem2*) and Tenascin C (*Tnc)* with downregulated genes represented by Aldolase, Fructose-Bisphosphate B (*Aldob*), and Zinc Finger BED-Type Containing 6 (*Zbed6*) (Figure 2A). Genes were selected based on their expression changes and later referred to by their function in heart tissue regulation. This set of genes has been evaluated using qPCR with only *Serpinb1c, Nupr1, Trem2, Tnc* and *Aldob* expression confirmed to be significantly different (Figure 2B).

### 2.3. Correlation of Funtional PET/CT (SYN1, [^18^F]-FDG) and MRI with Molecular Expression Markers Levels in Myocardial Infarction Model

**Figure 3:**
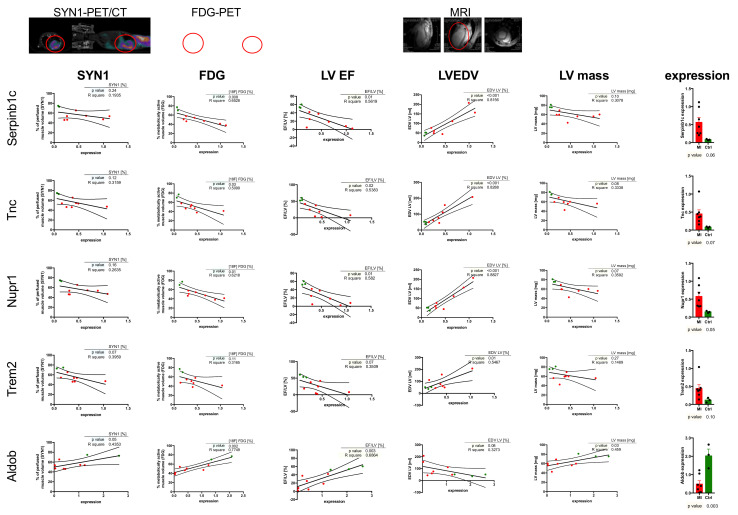
Correlation between gene expression and heart perfusion, metabolic and functional parameters. Above the graphs: SYN1-PET/CT scans of mice in the abdominal and lateral positions. FDG-PET-PET scans of the same mouse in the abdominal and lateral positions. MRI imaging— heart scans in three planes. Red circles indicate visible losses of marker accumulation and places of myocardial impairment. SYN1, [^18^F]-FDG and MRI parameters (LV EF, LVEDV, LV mass) have been correlated with expression pattern of selected genes (*Serpinb1c, Nupr1, Trem2, Tnc* and *Aldob*). For each correlation, a linear regression analysis was performed. Charts include 95% CI (dashed line). Values *p* ≤ 0.1 are considered to be significant. Detailed data discussion in the main text.

After determination of expression pattern, we attempted to introduce a combinatorial approach. The data from PET/CT scans based on SYN1 and [^18^F]-FDG, together with left ventricular ejection fraction (LV EF), left ventricle end-diastolic volume, and left ventricular mass obtained from MRI measurements were correlated with gene expression selected in the previous step, namely: *Serpinb1c*, *Nupr1*, *Trem2*, *Tnc* and *Aldob* (Figure 3). Several observations could be driven by this comparison.

First, expression of the selected marker genes that were upregulated after MI (*Serpinb1c, Nupr1, Trem2, Tnc)* was inversely proportional to the size of the infarct and its functional impact on myocardial function. Among these, left ventricular ejection fraction (LV EF) has been inversely proportional to the expression of genes upregulated after MI (increased expression -> decreased LV EF) and directly proportional to the *Aldob* downregulated after MI (decreased expression -> decreased LV EF). Left ventricle mass calculated from MRI experiments followed a similar pattern, although the correlation remained statistically significant only for *Serpinb1c, Tnc, Nupr1* and *Aldob*, showing diminished sensitivity of LV mass as a functional marker. Left ventricular end-diastolic volume (LVEDV) showed the opposite to LV EF correlations. Interestingly, LVEDV was the most coherent parameter out of the parameters used for analysis when taking into account the relevance of the correlation (R^2^ > 0.8 for *Serpinb1c, Tnc*, and *Nupr1*; with maintained statistical significance in all cases). MRI parameters, due to their direct relationship to the structural parameters of the heart muscle, provided the most precise data and correlations (as of R^2^), though the time and costs of the analysis remained significantly higher than with PET/CT.

PET/CT radiotracers evaluated in this work followed trends similar to LV EF (inversely proportional to the gene expression levels). In the case of [^18^F]-FDG, in the majority of the analysed genes, the significance of the correlation was maintained with exception of Trem2. What is important is SYN1, besides being considered a purely perfusion-based PET/CT radiotracer, showed a trend similar to [^18^F]-FDG and LV EF, with increased signal in postinfarction hearts, inversely correlating with genes upregulated after MI and directly correlating with downregulated genes in the MI group (Aldob), although these trends were statistically significant for Trem2 and Aldob only (*p* = 0.07 and 0.05, respectively).

### 2.4. Biodistribution Analysis of SYN1 in the Rat Model

**Figure 4:**
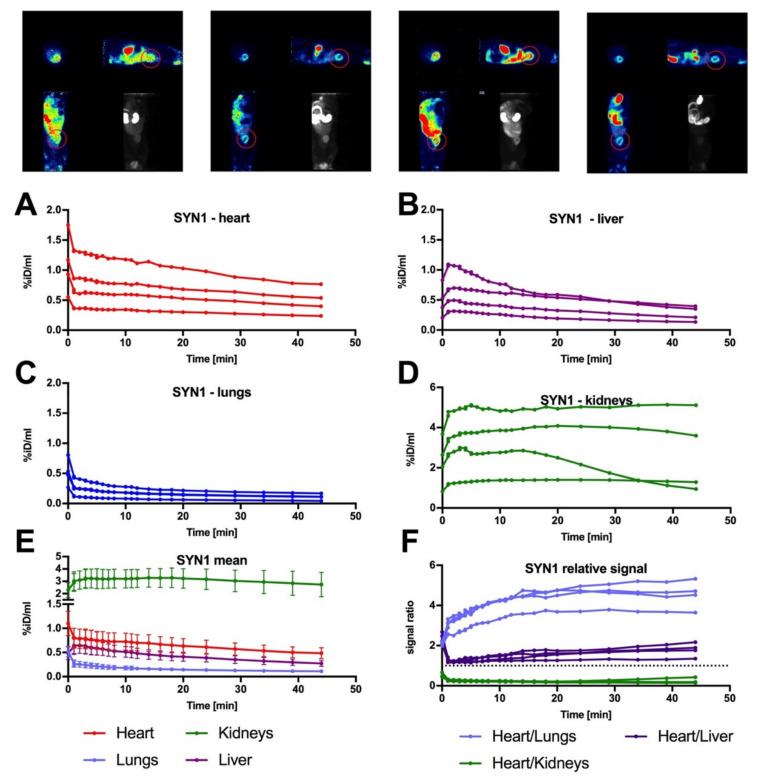
Biodistribution pattern of SYN1 in control rats. Above the graphs rats’ PET images of dynamic biodistribution of SYN1 are presented. Images represent all three planes at 20 min of color-coded images and a grayscale scan in the dorsal plane showing the average accumulation of the radiotracer in the body of each of the 4 animals. Red circles indicate exactly outlined myocardium on the lateral and dorsal planes. For analysis, signal was traced in 25 timepoints over 45 min for each of four animals (represented on the top of the figure) and collected for four different organs: heart, liver, lungs and kidneys (**A**–**D**). (**E**) Mean value with SEM for each organ. (**F**) Relative value of signal in the heart in relation to lungs, liver and kidneys. Dotted line represents 1:1 ratio.

Taking into consideration future clinical application for SYN1 as a perfusion radiotracer, we have performed a biodistribution analysis of the SYN1 presence in Wistar rats. In our analysis, we have selected four organs that remain crucial in terms of advanced PET imaging: heart, liver, lungs, and kidneys (Figure 4A–D for single distributions). The total signal collected from heart tissue was significantly higher (statistical data are not shown) in each analysed time point than the signal in lungs or liver, suggesting good specificity of the radiotracer (approximately four-times higher signal in the heart than in lungs and two-times higher than in the liver, Figure 4E,F). On the other hand, signal revealed in kidneys dominated over the heart intensity by approximately five-fold (Figure 4E,F). It remained high, with a tendency to decline over time, suggesting good renal clearance of SYN1, further supporting the positive biodistribution parameters of SYN1. No toxic effect was observed during and after biodistribution studies.

## 3. Discussion

Cardiac perfusion imaging is an effective supplement to metabolic studies in determining myocardial dysfunction [12]. The major difference is that it helps to understand the blood flow in coronary vasculature rather than depicting the activity of the muscle itself (compared to metabolic imaging). The examination is based on the assessment of the blood supply to the left ventricle muscle at rest and during stress, such as exercise. The blood supply is determined whether the impairment is in stable remission or temporary. Conclusions are drawn about the advancement of coronary artery disease or damage to the cells of the myocardium [9]. Full diagnostics has been used to classify the patient for treatment or to assess the effectiveness of the treatment. One of the major advantages of diagnostics with radioactive compounds is its noninvasiveness through the use of isotopic techniques such as positron emission tomography [13]. The availability of perfusion radiotracers is limited, but compounds based on fluorine-18 are gaining popularity because of its optimal half-life, allowing for precise planning of the diagnosis [8]. This timing allows imaging to be performed for prolonged cardiac activity tracking procedures. ^18^F is a very important solution not only because of its half-life but also because of the shortest range of positrons in the tissue compared to other currently used cardiac radionuclides, resulting in higher resolution of perfusion imaging [4]. Many research groups are looking for a new radiotracer for myocardial perfusion imaging. In our case, a [^18^F]-radiolabelled compound was employed. Initial functional evaluation was conducted using mice as laboratory animals according to 3R rules.

In this study, the [^18^F]-FDG metabolic radiotracer was used to provide a reference to the new fluorine-18-based SYN1 perfusion radiotracer. The primary aim of the research was to prove the validity of the use of the SYN1 radiotracer for research and future diagnostics of heart lesions. Both studied compounds showed an even distribution of radioactivity in the myocardium of control rodents and allowed the localization of damaged zones after induction of myocardial infarction with no difference between radiotracers observed (Figure 1). The multimodal approach with the simultaneous use of computed tomography techniques allowed for the precise determination of metabolic parameters and cardiac perfusion [14]. We present here that both [^18^F]-FDG and the SYN1 radiotracers had uniform distribution in the myocardial segments, while signal accumulation decreased in the upper parts of the left ventricle walls compared to the rest of the heart segments in control rodents (Figure 1). With imaging of postinfarction mice, the situation was the opposite, as the first segments corresponding to the periapical region showed less isotope uptake compared to the upper ones. Interestingly, when groups of animals were distributed according to MI size, the highest heterogeneity between segments was observed in the medium-sized MI. This is because in medium-sized infarction, segments that remain not affected act in a compensatory state, and relative signal (both perfusion- and metabolic-based) increases. In the group of large infarction, all parts of the left ventricle might be affected, thus compensation is less efficient and the relative signals in each segment are more coherent. This observation brings an interesting conclusion of good signal specificity of the tested SYN1 radiotracer, even when used in relatively minuscule animal models such as mice, compared to larger MI models used for similar observations [15].

We have evaluated the size of the metabolically and perfused active area in normal and postinfarction mice. Considering the remodelling of the heart and its enlargement after myocardial infarction [16], it can also be observed that the SYN1 radiotracer showed similar areas in terms of volume in normal and postinfarction mice compared to the [^18^F]-FDG metabolic radiotracer. Depending on the size of the lesion in each individual, SYN1 showed a similar accumulation in the relevant segments. The precise division of the mouse heart showed the validity of the use of SYN1 in the imaging of myocardial infarction.

In the next step, we tested molecular characteristics of the heart after infarction. Out of 11 selected genes, 5 were confirmed by qPCR. These were Serpinb1c, Nupr1, Trem2, Tnc upregulated in MI animals and Aldob, downregulated in the MI group (Figure 2B). Serpinb1c is also known as the Leukocyte elastase inhibitor C, which regulates the activity of the neutrophil proteases and plays a role in inflammation, limiting the response of CASP 1 and 4 [17]. On the other hand, Trem2 is a major marker of a novel macrophage population, different from inflammatory macrophages found in atherosclerotic aortas, acting in the diseased tissue calcification process [18]. It seems that both Serpinb1c and Trem2 are markers of advanced remodelling, neatly corresponding to and correlating with the tissue phenotype observed in this study.

A less known although interesting role is played by Nupr1. Here, we found it upregulated in the MI group. Research concerning the role of Nupr1 focuses on mitochondrion-related endothelial cell apoptosis. It is believed that Nupr1/Chop signalling may be a potential therapeutic target in drug-induced cardiovascular toxicity [19]. Here, Nupr1 could be a marker of endothelium disruption, although its presence in late MI in the postinflammatory heart tissue remains elusive.

The remaining two genes play a far more recognized role in MI. Tenascin C has an established role in the cardiomyocyte adhesion process and is defined as a marker in diabetes [20]. Furthermore, increased myocardial expression of Tnc was associated with worse long-term outcomes in dilated cardiomyopathy patients [21], which share some similarities when compared to long-term remodelling after ischemic injury. Finally, Aldolase B is a long-known serum marker, that increases in the peripheral blood after MI upon its release from injured cells. Its levels decrease with time. In our study, expression levels of Aldob were decreased long after MI incidence. This is a result of diminished amounts of healthy cardiac tissue.

In the current work, we aimed to define correlations between different physiological methods of cardiac muscle imaging and the molecular markers discussed above. As presented in Figure 3, these correlations or trends have been maintained for all used parameters, reciprocally strengthening their diagnostic value. Nevertheless, these parameters should be used in cohesive manners as they provide complementary, not interchangeable information. Although MRI-based measurements are precise, they often are problematic in terms of their fast diagnostics and cost-effectiveness. [^18^F]-FDG has become a gold standard of PET/CT-based metabolic imaging, providing information about metabolically active muscle. As a glucose-based radiotracer it correlates well with Aldolase B, declining in expression and sharing the same metabolic basis. SYN1, as a perfusion-based radiotracer, showed similar trends to [^18^F]-FDG in the proposed gene setup, although this was not readily expected. SYN1 proved similar, with a direct correlation with *Aldob* expression and inverse correlation with *Trem2* expression (while [^18^F]-FDG did not show correlation efficiency in this case), showing SYN1 has functional differences to [^18^F]-FDG, potentially further widening the descriptive nature of the PET/CT radiotracers. This is especially interesting when taking into account perfusion-dependent characteristics of *Trem2* expression (present on macrophages, late heart remodelling marker). This, however, together with the observed lack of statistical significance in the case of some of the molecular markers should be clarified by enlarging study groups in which tendencies were observed.

Based on mouse studies, the SYN1 radiotracer was tested in Wistar rats. Dynamic imaging was performed to determine the biodistribution of the compound in the animal body. Accumulation of the compound in the heart, liver, kidneys, and lungs during PET/CT imaging was investigated. SYN1 radiotracer exhibited a proper uptake in healthy hearts by showing the exact outline of the myocardium. The highest uptake levels were found in tissues involved in the process of excretion due to efficient renal clearance (as presented by the maintenance of high heart/kidney ratio), which is crucial for radioactive compounds [22].

There are several limitations of our study. First, the groups of animals used were limited. In some cases (especially in the SYN1 group) increased animal number would provide a better discernible effect in terms of the infract size and PET/MRI/gene expression correlations. This concerns the control group as well. Still, comparison between [^18^F]-FDG and SYN1 could be performed and discussed. In order to complement our knowledge, next, large scale preclinical studies with larger rodents should be planned. Furthermore, our study lacks direct angiography studies—this however is another limitation of early phase studies with small animal models. Investigating a more clinically relevant model (dogs, pigs) would surely add the resolution of the SYN1 radiotracer’s efficiency in the detection of the injured heart. Although being a perfusion radiotracer, not a metabolic one, SYN1 still shows tendencies and correlations similar to radiotracers more targeted to such applications ([^18^F]-FDG) and parameters with much longer analysis time (MRI). Using perfusion-relevant resolution and techniques to define the function of SYN1 would be a much more suitable technique with more reliable efficiency.

Taken together, our study introduces a new fluorine-based perfusion radiotracer for PET/CT imaging. Having in mind the tremendous need for new, reliable, time-efficient cardiovascular imaging possibilities, we see an immense oppertunity in introducing SYN1 into the market after the next stage of large-animal preclinical studies.

## 4. Materials and Methods

### 4.1. Animals and Experimental Design

The Local Ethical Committee for Animal Research at Poznan University of Life Sciences and The Ist Local Ethical Committee for Animal Research in Warsaw both approved protocol for the experiments performed in the mice post-infarction heart model, while the rat experiments were approved by the latter committee only (no experiments with rats were performed in Poznan). All animal experiments were performed under relevant guidelines and regulations.

The experiments were carried out in NOD-SCID mice (NOD, CB17-*Prkdc^scid^*/NCrCrl, Charles River, UK) and Wistar rats (Cmd: WI (WU), Mossakowski Medical Research Centre Polish Academy of Science, Poland). PET/CT imaging was performed on mice divided into control (*n* = 2) and post-infarction (*n* = 7) groups and rats (*n* = 4).

**Figure 5:**
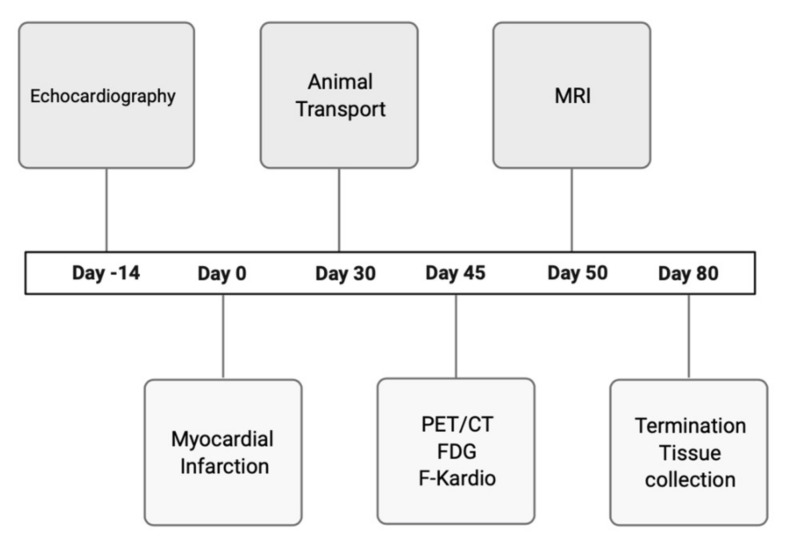
Experimental design and timeline of the procedure.

The study protocol for mice is presented in Figure 5.

SYN1 and [^18^F]-FDG radiotracers were used in PET/CT scan in order to evaluate the perfusion and metabolism in the myocardium. Assessment of cardiac hemodynamic parameters was performed using MRI in order to determine how the cardiac infarction influenced the left ventricular region and its subsequent contractility. A 7T Bruker Biospec scanner (70/30 USR, Bruker, Biospin, Ettlingen, Germany) was used with a set of coils—only the surface coil and transmitted cylindrical radiofrequency volume coil (10 mm and 8.6 cm inner diameter, respectively) were applied. Anaesthetized mice (1.5% isoflurane in a mixture of oxygen and air) were placed in an MR-compatible bed. Based on pilot scans, geometry for the four-chamber view of the long axis of the heart has been set. Using four-chamber view, a set of short-axis scans was performed with IntraGateFLASH protocol covering all ventricles’ volumes. Protocol parameters used in the experiment: echo time = 3 ms, repetition time = 10 ms, number of repetitions = 120, field of view = 25 mm × 25 mm, slice thickness = 0.9 mm, spatial resolution = 0.13 × 0.13 mm per pixel. Each heartbeat cycle contained 15 images. In order to improve image quality, a gating system was used.

Reconstructed MRI data in DICOM format were further analysed to perform a calculation of end-systolic and end-diastolic volume of heart ventricles. For each short-axis scan, inner edges of the ventricle were outlined manually in OsiriX DICOM Viewer (Pixmeo SARL, Bernex, Switzerland). End-systolic and end-diastolic images were defined and used further for the calculation of ventricles volume.

### 4.2. Induction of Myocardial Infarction

Myocardial infarction was induced in twelve-week-old NOD-SCID mice by ligating the left anterior descending coronary artery under isoflurane anaesthesia with 100% oxygen ventilation. Subsequently, the chest was closed and sutured, and the mice could recover.

### 4.3. Echocardiography 

The animals were anesthetized by intraperitoneal injection of xylazine/ketamine solution. Long- and short-axis measurements of the left ventricle area, in both systole and diastole, were performed using a GE Vivid 7 high-resolution ultrasound scanner equipped with a M12L linear transducer (GE Healthcare, Chicago, IL, USA). The measurements were used to calculate left ventricle fractional area change (LVFAC) in order to monitor left ventricle remodelling. Calculations were performed using the formula below:

$$\mathrm{LVFAC}=\frac{\mathrm{LVEDA}-\mathrm{LVESA}}{\mathrm{LVEDA}}\times 100\%$$

LVFAC—left ventricle fractional area change.

LVEDA—left ventricle area in diastole.

LVESA—left ventricle area in systole.

### 4.4. Radiochemistry

SYN1 was synthesized according to well-established ^18^F-labelling procedure based on nucleophilic substitution reaction, in which the active leaving group of the precursor is replaced by ^18^F^−^. The fluoride-18 was produced in the ^18^O(p,n)^18^F nuclear reaction on the Eclipse, Siemens cyclotron. Then, the obtained ^18^F^-^ was loaded on a preactivated ion-exchange column (QMA), and eluted to reactor with kryptofix-2.2.2./potassium carbonate solution. Next, azeotropic removal of water and acetonitrile was conducted. Afterwards, the precursor dissolved in dichloromethane was added to the reactor to carry out the labelling reaction. Once the reaction was finished, the crude product was diluted with acetate buffer and was purified using semi-preparative HPLC. The final product was formulated using 0.9% saline solution and sterilized by filtration. The identity of the final product was confirmed using an analytical HPLC system with radiochemical purity greater than 95%. The patent application covering the compound synthesis and application was submitted.

### 4.5. Acquisition Protocol [^18^F]-FDG and SYN1

NOD-SCID mice were administrated via the tail vein with [^18^F]-FDG (10.5 +/− 2.0 MBq) in a total volume of 150 µL. Then, 60 min after injection, the animals were placed in an induction chamber for initial anaesthesia (isoflurane 3.5–4.0%), weighted, and transferred to the scanner bed for general anaesthesia through isoflurane (1.5–2.0%). In the case of imaging SYN1, NOD-SCID mice (26.7 +/− 3.9 g) and Wistar rats (382.0 +/− 8.7 g) were injected via the tail vein (7.8 +/− 2.0 MBq for mice, 11.7 +/− 0.5 MBq for rats). Static imaging of mice was performed 30 min after compound injection, while dynamic imaging was performed immediately after injection into anaesthetized rats. We used a trimodal small-animal scanner Albira Si PET/SPECT/CT Preclinical Imaging System (Bruker, Billerica, MA, USA).

### 4.6. PET/CT Image Fusion and Data Analysis

Data were reconstructed using the built-in program Albira software and analysed in PMOD v4.02. Physiological colour-coded PET scans were applied on anatomical, grey-coded CT ones to accurately locate the radioactive compounds. PET/CT images were visually interpreted and regions of interest (ROIs) corresponding to the examined organs were manually drawn to determine their accumulated radioactivity content, which was then converted to a percentage of injected dose per volume of tissue (%ID/mL).

### 4.7. Heart Tissue Collection

The mice were terminated on the 8th day of the experiment by cervical dislocation. Their isolated hearts were washed three times with a 0.9% NaCl solution, and the left ventricles were isolated and stored in RNALater (Ambion, Foster City, CA, USA) solution at –80 °C or in liquid nitrogen.

### 4.8. Sample Preparation and Gene Expression Analysis

Left ventricle tissue was cut into small pieces, placed in 1 mL of TRI Reagent (Sigma-Aldrich, St. Louis, MO, USA) with protease inhibitors and homogenized (Homogenizer Workcenter T10 Basic ULTRA-TURRAX^®^, Whitestown, Ireland). Finally, a standard TRIzol protocol was applied for RNA isolation. RNA yield was determined using a Nanodrop 2000 (Thermo Scientific, Waltham, MA, USA), and its quality was assessed using standard 1.5% agarose gel electrophoresis.

Total RNA was further purified using a TURBO DNA-free kit (Thermo Scientific, Waltham, MA, USA). Afterwards, 1 μg of each RNA sample was used for reverse transcription to obtain cDNA template for qPCR (iScript- Bio-Rad, Hercules, CA, USA). The real-time PCR quantitative reactions were performed using a CFX Connect Real-Time PCR Detection System with a SsoAdvanced Universal SYBR Green Supermix (Bio-Rad, Hercules, CA, USA). Based on the available literature, three reference genes were selected: *ActB*, *Tbp* and *Hprt*. The results were normalized using mentioned reference genes, and the expression of selected genes was calculated using GeNorm algoritm.

The qRT-PCR sequence primers are listed in Table 1.

### 4.9. Analysis of Next-Generation Sequencing

Quality of obtained data was checked by FastQC online tool- https://www.bioinformatics.babraham.ac.uk/projects/fastqc/ version 0.11.9 (Babraham Bioinformatics, Cambridge, UK) version 0.11.9 [23] designed for high-throughput sequence data. Before mapping, all adapters were trimmed with usage of Awk software (version 3.1.7). Later on, reads were mapped by STAR (version 2.5.3a) [24] to human and mouse genomes (*Homo*_*sapiens*.GRCh38.89 *Mus*_*musculus*.GRCm38.89.gtf) and counted by Rsubread (version 2.0.0) [25], a Bioconductor (version 3.10) software package that provides high-performance alignment and read counting functions for RNAseq. Normalization and differentially expressed genes analysis (DEG) was performed using edgeR package (version 3.24.3) [26]. The functional enrichment analysis was conducted using topGO tools (version 2.38.1) [27] of Bioconductor package. We tested the enrichment of GO terms with differentially expressed genes [28] (with adjusted *p*-value < 0.05 and Log2FC > 0.5) using Fisher’s exact test. All figures in this paper were created using the ggplot2 (version 3.2.1) R package [29].

### 4.10. Statistical and Correlation Analysis

For the real-time PCR analysis, the experimental samples were run in duplicate. Values are shown as the mean ± SEM. Statistical analysis was performed using GraphPad software. Statistical significance was evaluated with Mann–Whitney, ANOVA tests, simple linear regression between two continuous variables and Spearmans test for measuring linear and nonlinear relationships between two continuous variables. Values with *p* ≤ 0.1 were considered to be statistically significant.

## Figures and Tables

**Table 1:** List of the primers and their detailed specification used in the mouse gene expression study.

ID	Species	Gene	Product (bp)	R^2^	Efficiency	Cq	Cm (°C)
qMmuCID0008546	*M. Musculus*	*Wisp2*	124	0.9999	100%	23.11	88
qMmuCID0025322	*M. Musculus*	*Timp1*	149	0.9996	99%	20.81	85
qMmuCID0017767	*M. Musculus*	*Serpinb1c*	95	0.9999	100%	24.14	80
qMmuCID0018300	*M. Musculus*	*Mmp12*	100	0.9999	96%	24.12	80.5
qMmuCID0024737	*M. Musculus*	*Serpina3n*	179	0.9996	96%	25.3	86
qMmuCED0001658	*M. Musculus*	*Nppa*	126	0.9995	102%	27.47	86.5
qMmuCID0007257	*M. Musculus*	*Nupr1*	152	0.9993	98%	21.18	84.5
qMmuCID0020213	*M. Musculus*	*Trem2*	125	0.9994	96%	22	79.5
qMmuCED0039716	*M. Musculus*	*Aldob*	112	0.9998	95%	28.82	79
qMmuCID0012108	*M. Musculus*	*Clstn3*	97	0.9998	98%	24.92	87
qMmuCED0040037	*M. Musculus*	*Zbed6*	94	0.9932	103%	20.4	79.5
qMmuCID0005706	*M. Musculus*	*Tnc*	87	0.9994	98%	20.12	80.5
qMmuCID0006635	*M. Musculus*	*Ddr1*	63	0.9994	96%	21.26	79
qMmuCID0018270	*M. Musculus*	*Ddr2*	120	0.9997	96%	19.55	84
qMmuCID0016589	*M. Musculus*	*Atf2*	86	0.9997	101%	21.07	82
qMmuCID0007732	*M. Musculus*	*Atf5*	141	0.9991	100%	27.8	89
qMmuCID0005679	*M. Musculus*	*Hprt*	70	0.9998	94%	19.17	77
qMmuCID0040542	*M. Musculus*	*Tbp*	102	0.9997	97%	21.36	84.5
qMmuCED0027505	*M. Musculus*	*ActB*	109	0.9996	101%	12.52	84

## Data Availability

All the data and analyses are available upon request.

## Supplementary Materials

The following are available online at https://www.mdpi.com/article/10.3390/ijms222212591/s1, Figure S1: Comparison of molecular imaging of control and MI mice; Figure S2: Summary of the expression data between MI and control groups.
