# Supplementary material for: Multiparametric Evaluation of Post-MI Small Animal Models Using Metabolic ([18F]FDG) and Perfusion-Based (SYN1) Heart Viability Tracers
Source: Int J Mol Sci. 2021 Nov 22;22(22):12591. doi: 10.3390/ijms222212591 (PMC8619497; doi:10.3390/ijms222212591)
Supplement: Supplementary file 1 [file ijms-22-12591-s001.zip › ijms-1432702-supplementary.pdf]

International Journal of
Molecular Sciences

MDPI

Supplementary Material

Multiparametric evaluation of post-MI small animal models using metabolic ([18F]FDG) and perfusion-based ([18F]Fkardio) heart viability markers

Tomasz Jan Kolanowski [1,†], Weronika Wargocka-Matuszewska [2,†], Agnieszka Zimna [1], Lukasz Cheda [2], Joanna Zyprych-Walczak [3], Anna Rugowska [4], Monika Drabik [5], Michał Fiedorowicz [5], Seweryn Krajewski [6], Łukasz Steczek [6], Cezary Kozanecki [6], Zbigniew Rogulski [2], Natalia Rozwadowska [1] and Maciej Kurpisz [1,*]

Citation: Kolanowski, T.J.; Wargocka-Matuszewska, W.; Zimna, A.; Cheda, L.; Rugowska, A.; Drabik, M.; Fiedorowicz, M.; Krajewski, S.; Kozanecki, C.; Rogulski, Z.; et al. Multiparametric Evaluation of post-MI Small Animal Models Using Metabolic ([^{18}F]FDG) and Perfusion-Based (SYN1) Heart Viability Tracers. *Int. J. Mol. Sci.* **2021**, *22*, 12591. https://doi.org/10.3390/ijms222212591

Academic Editor: Manfredi Rizzo

Received: 7 October 2021
Accepted: 16 November 2021
Published: 22 November 2021

Publisher's Note: MDPI stays neutral with regard to jurisdictional claims in published maps and institutional affiliations.

Copyright: © 2021 by the authors. Licensee MDPI, Basel, Switzerland. This article is an open access article distributed under the terms and conditions of the Creative Commons Attribution (CC BY) license (http://creativecommons.org/licenses/by/4.0/).

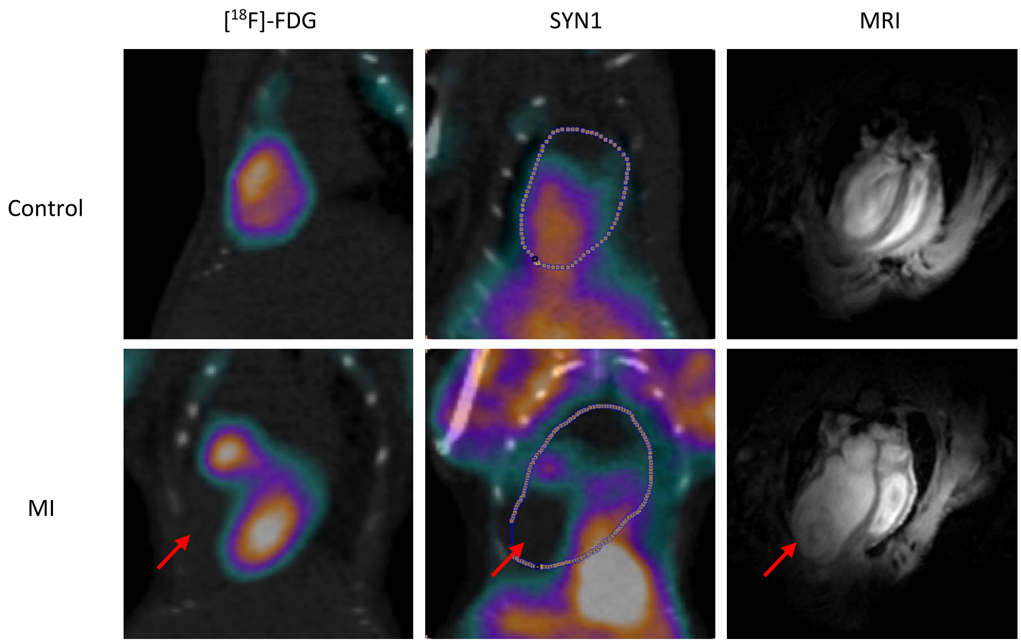

Figure S1. Comparison of molecular imaging of control and MI mice.

Control mouse (top row) and post MI mouse (bottom row). Note the difference in the loss of [18F] -FDG and SYN1 uptake in the apex region. MRI imaging confirms the damage of the heart muscle left ventricle, which is also visible in the images. The size of post MI heart is bigger due to the organ remodeling.

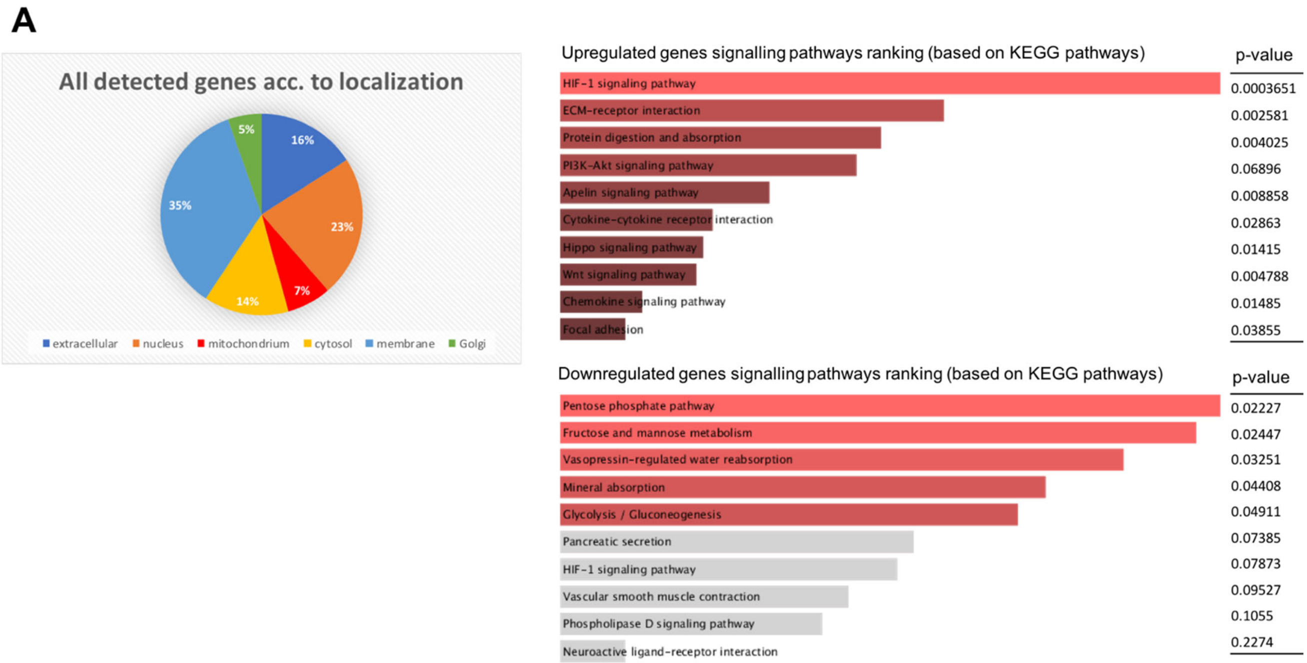

Figure S2. Summary of the expression data between MI and control groups

A. Localization of the differentially expressed genes (DEG) shows that the major changes occur in the extracellular and membrane compartments, accompanied with the nuclei expression changes.

B. B. Major up- and downregulated signaling pathways in post MI group. Signaling pathway ranking is based on reversed p-values. P-values are presented next to the chart.
